# Factors influencing entrepreneurial intention of university students in Yemen: The mediating role of entrepreneurial self-efficacy

**DOI:** 10.3389/fpsyg.2023.1111934

**Published:** 2023-01-25

**Authors:** Nabil Al-Qadasi, Gongyi Zhang, Mohammed Ali Al-Awlaqi, Ali Saleh Alshebami, Ammar Aamer

**Affiliations:** ^1^School of Business and Management, Jilin University, Changchun, Jilin, China; ^2^Faculty of Commerce and Economics, Sana’a University, Sana’a, Yemen; ^3^School of Business, Lebanese International University, Sana’a, Yemen; ^4^Applied College, King Faisal University, Alahsa, Saudi Arabia; ^5^College of Professional Studies, Northeastern University, Toronto, ON, Canada

**Keywords:** entrepreneurial intentions, personality traits, environmental factors, situational factors, university students, Yemen

## Abstract

While entrepreneurship is believed to play a crucial role in economic growth and job creation in various parts of the world, particularly in developed countries, the key factors enhancing entrepreneurship behavior and intention in developing countries still need to be discovered. Therefore, this study examines the influence of personality traits and environmental and situational factors on the development of entrepreneurial intention among young students in Yemen. Data were collected through a survey responded to by 487 final-year university students from two universities (public and private) in Yemen. The study’s hypotheses were tested using structural equation modeling (SEM). The study reveals that personality traits of the need for achievement (nAch) and locus of control (LoC) positively correlate with entrepreneurial self-efficacy (ESE) and entrepreneurial intention. Instrumental readiness positively correlates with ESE but not with entrepreneurial intent. The situational factors show a positive association with entrepreneurial intention but not ESE and a positive relationship between ESE and entrepreneurial intention. Furthermore, the study’s findings show that ESE partially mediates the relationship between the nAch, LoC, instrumental readiness, and entrepreneurial intention. However, ESE did not mediate the relationship between situational factors and entrepreneurial intention. The study suggests that situational factors can influence entrepreneurial intention among Yemeni students and provide several recommendations to academicians and policymakers.

## 1. Introduction

Throughout human history, entrepreneurship has long been associated with economic growth and social stability (Lent et al., 2002). Entrepreneurs are economic agents who bring fresh ideas, creative solutions, and new job opportunities to help spin the economy’s wheel (Al-Awlaqi et al., 2021). However, the uncertainty of the business future, political crises, and the accompanying global economic changes have shocked many. The stiff competition among organizations and global economic insecurity continue to push businesses to cut operational costs and restructure work processes (Lüthje and Franke, 2003). This eventually leads to laying off employees and puts more pressure on governments because of the unavailability of jobs and a high unemployment rate. The negative consequences resulting from laying off employees necessitate the development of alternatives to substitute salaried employment and allow the creation of new job opportunities for the betterment of individuals’ life. One of these alternatives is the development of entrepreneurship and the Small to Medium Enterprises (SME) sector, as they contribute significantly to the economy’s development and growth by increasing job opportunities, alleviating poverty, increasing individual empowerment, and increasing self-reliance and efficacy (Elnadi and Gheith, 2021; Aljarodi et al., 2022; Al-Mamary and Alshallaqi, 2022; Alshebami and Seraj, 2022).

Despite the belief that entrepreneurship provides numerous benefits to various stakeholders in society, particularly the youth, it is believed that many challenges are limiting its growth and development. These challenges may differ depending on the context in which business activities are carried out. For example, social relationships, cultural climate, administrative complexities, access to resources, economic conditions, institutions, and available infrastructures may significantly encourage or discourage the development of entrepreneurship among individuals, particularly entrepreneurial intention (IntEnt) and behavior (Kristiansen, 2002; Kristiansen and Indarti, 2004; Gurbuz and Aykol, 2008). This means that societies with a stable economic situation, a supportive culture, developed infrastructure, and encouraging formal and informal institutions and laws may encourage people to start their businesses. Meanwhile, countries with excessive administrative complexities, limited access to business information, and capital are barriers to a business establishment (Akanbi and Ofoegbu, 2011).

Even though environmental factors and other condition factors (economic-political conditions) can impede entrepreneurship growth, a significant question remains as to what causes some people to behave as entrepreneurs while others do not (Mitchell et al., 2007; Gerba, 2012). This directs our research toward investigating other factors, such as people’s psychological factors, which may influence entrepreneurial behavior and intention. Many psychological traits may contribute to individuals’ IntEnt; however, previous research indicates that specific characteristics, such as the need for achievement (nAch), locus of control (LoC), and entrepreneurial self-efficacy (ESE), contribute the most (Sengupta and Debnath, 1994; Mitchell et al., 2007; Alshebami, 2022).

The nAch is the individual initiative to act to achieve success and create businesses with competitive advantages (Ida Ketut, 2019). On the one hand, individuals with a high nAch are more likely to develop IntEnt (Schaper et al., 2010; Naushad, 2018; Ndofirepi, 2020). On the other hand, those with a low nAch tend to develop insufficient competencies and inspirations (Kristiansen and Indarti, 2004). Furthermore, LoC is another critical key psychological factor influencing one’s decision to start a business. The term LoC dates back to Rotter (1966). The LoC refers to an individual’s belief that they control their actions and daily events (Arkorful and Hilton, 2021; Alshebami and Seraj, 2022). People with a high internal LoC have more IntEnt, whereas those with a high external LoC have less entrepreneurial behavior (Lefcourt, 2014; Vodă and Florea, 2019).

Furthermore, ESE contributes to the development of IntEnt. ESE is the extent to which individuals believe they can carry out specific business tasks (Bullough et al., 2014; Alshebami, 2022). People with high self-efficacy levels are more likely to complete their tasks successfully (Zhao and Wibowo, 2021). As a result, people increase self-resilience when running their entrepreneurial businesses (Nisula and Olander, 2020). In summary, the previously mentioned three psychological traits, environmental factors, and other economic-political status-related factors may all play a significant role in the development of IntEnt among individuals, particularly young ones (Phan et al., 2002; Kennedy et al., 2003; Kristiansen and Indarti, 2004).

Despite numerous studies reporting the effect of the previously mentioned psychological, environmental, and situational factors on individuals’ IntEnt, it is not widely known whether the founding conditions or personality characteristics drive students’ IntEnt and careers toward establishing their businesses (Lüthje and Franke, 2003). Few studies have been conducted to examine IntEnt among students, particularly in developing countries (Lüthje and Franke, 2003; Gerba, 2012; Alshebami, 2022). Psychological, environmental, and situational factors, as well as their influence on one’s IntEnt, have been used as separate factors in previous studies (Sesen, 2013). Only a few studies attempted to investigate the influence of the three selected factors in the study as groups of factors in one IntEnt model (Davidsson, 1995; Lüthje and Franke, 2003; Al-Qadasi et al., 2021).

Some authors argue that investigating one group of factors in isolation from the others may result in misleading and incorrect findings Anjum et al. (2020) and create a research gap that must be addressed. Therefore, in this study, we combine psychological, environmental, and situational variables/factors into a single model and examine their effect on IntEnt and the impact of ESE. Additionally, and as stated earlier, the studies in the extant literature addressed the selected above factors among young individuals, particularly students, who are limited in developing countries, particularly in the Yemeni context. As a result, this study focuses on Yemen, one of the poorest Arab countries with numerous economic and political challenges. Entrepreneurship and entrepreneurial activity at the country level are in a constant state of dynamism. Yemeni youth are constantly developing new business ideas, taking steps to get one off the ground, starting a new business, and running an existing business motivated by earning a living. Since 2011, Yemen has witnessed and is living the most challenging crisis. Millions of people lost their jobs because of the war and the difficult circumstances it brought. Most production activities have stopped, and the private sector has laid off most of its employees. Almost half of the country’s population has no income and is struggling to earn a living (Yemen’s population is around 30 million). According to the international poverty rate, Yemen has a poverty rate of about 18.8%, while the national rate is about 48.6%. The country’s recent war caused the economy to contract by 39% and created numerous economic challenges (World Bank, 2019; Alshebami and Alzain, 2022). Almost every sector in Yemen has been impacted by the ongoing war and internal conflicts, including the SME sector (Al-Awlaqi and Aamer, 2022).

In addition to the challenging conditions in the country, entrepreneurs in Yemen lack the ability, training, and support to develop their entrepreneurial skills, mainly female (Ahmad, 2011). Yemeni entrepreneurs need financial support for technical training, coaching services, and development programs (Alshebami and Alzain, 2022). Yemen is also ranked first out of 55 economies in terms of the percentage of adults who believe self-employment is a good career option (Stevenson et al., 2010). Accordingly, it is critical to creating an environment for entrepreneurs in Yemen, particularly young entrepreneurs. This can be accomplished by directing the government and other stakeholders’ efforts toward improving and developing educational programs, necessary infrastructures, laws and regulations, and financial support to eventually lead to more self-employment and small business creation (Lüthje and Franke, 2003). To improve the development of entrepreneurial programs in the country, policymakers must first understand the key factors and elements that motivate individuals to start small businesses (Scott and Twomey, 1988).

This research aims to determine how some psychological/personality traits and environmental and situational factors can motivate IntEnt among potential Yemeni entrepreneurs (university students). It also intends to investigate the role of ESE in mediating the relationship between IntEnt and the previously mentioned factors. The study has a threefold contribution to the entrepreneur field: *First*, little is known about the factors that motivate people to engage in entrepreneurial activity, particularly in adversity-stricken societies. This study identified several personality traits and environmental and situational factors that may influence one’s IntEnt. *Second,* the study looks into the role of ESE in mediating the relationship between selected personality traits and environmental and situational factors, and one’s IntEnt. *Third,* this study focuses on situational factors – the political-economic crisis – and its effect on one’s IntEnt. Despite the large body of empirical studies measuring IntEnt in stable and robust environments, very few studies tested entrepreneurial career intentions in challenging contexts.

The study is organized as follows: after the introduction, the literature review and hypothesis development are discussed in the second section. The third and fourth sections discuss research methodology and data analysis. The fifth and sixth sections then discuss the discussion and conclusion.

## 2. Literature review and hypotheses development

### 2.1. Theoretical foundation

IntEnt is the first stage and the most crucial trigger of entrepreneurial behavior (Ruiz-Palomino and Martínez-Cañas, 2021); it is a psychological process that has been developed and tested by several researchers in social, personality, and organizational psychology (Stappers and Andries, 2022). Entrepreneurship literature shows robust debate among four major theories that explain IntEnt. These four theories include Shapero (1982) theory of the entrepreneurial event, Ajzen (1991) Theory of Planned Behavior, Davidsson (1995) economic-psychological model, and Lüthje and Franke (2003) model. These theories founded theoretical frameworks of any endeavor to understand IntEnt behavior.

The entrepreneurial event (EE) theory suggests three main factors that could affect an individual’s decision to start a new business: perceived desirability, perceived feasibility, and propensity to act upon opportunities. Perceived desirability measures how attractive is starting a new business to the individual. Perceived feasibility measures an individual’s perception of his ability to start a business. The propensity to act is “the disposition to act on one’s decisions” (Krueger et al., 2000).

The theory of planned behavior (TPB) assumes that three distinct attitudinal antecedents of intention determine an individual’s intention and behavior: the subjective norm, perceived behavioral control, and attitude toward the behavior (Ajzen, 1991). Attitude toward the behavior describes how individuals evaluate the performance of a specific behavior either positively or negatively. Subjective norms are the effect of the surrounding social groups on the decision of an individual to perform or not perform a particular behavior. The third antecedent of intentions is perceived behavioral control, which reflects a belief in an individual’s ability to start a new business.

Davidsson (1995) devolved an economic-psychological model to test the effect of economic and psychological factors on an individual’s intention to start a business. According to Davidsson’s model, an individual’s conviction is the primary determinant of IntEnt. This conviction is based on (i) general attitudes (need to change, achievement, autonomy, competitiveness, and money orientation), (ii) domain attitudes (payoff, social contribution, and know-how), and (iii) the current situation (current employment status). In their study, Guerrero et al. (2008) report that the last relevant IntEnt model integrated previous Ajzen (1991) TPB and Shapero (1982) theory of the EE is Davidsson’s model. However, in a later study, Lüthje and his colleague proposed a model dedicated to testing the influence of some personality traits and a set of contextual factors on one’s intention to start a business (Lüthje and Franke, 2003), which we discuss below.

In their study, Lüthje and Franke (2003) structural a model of IntEnt; the authors combined two factors, personality traits and contextual variables, to model students’ IntEnt. Personal traits are responsible for the individual attitude, while contextual variables are considered environmental factors that could support or undermine IntEnt. According to Nabi et al. (2010), Lüthje and Franke Model (LFM) provides a robust framework for assessing the antecedents of IntEnt. Previous research frequently used this model to investigate the personal and environmental determinants of IntEnt (Kristiansen and Indarti, 2004; Schwarz et al., 2009; Sesen, 2013; Al-Qadasi et al., 2021). Besides the previous discussion, Liñán and Fayolle (2015) state that despite the broad applicability of intent models, there is still more research to be done to understand better how the context reacts with the individual in determining the IntEnt.

Although these theories are well established in the literature, each theory is specialized and focuses on one aspect rather than the other. These theories are specialized in behavioral, psychological, sociological, or personal characteristics. This raises the importance of combining different factors from different dimensions or aspects. This would help understand the mutual and dynamic effects of these groups of factors. We developed an integrated model to investigate IntEnt. This model combines personality, contextual, and condition factors in a unique social-economic context. These factors and hypotheses development are discussed below.

### 2.2. Personality factors

As we explained in the previous section, major IntEnt models show that attitudes, personality traits, and contextual factors have a directive or dynamic influence on one’s behavior to be an entrepreneur. Personality traits are closely related to intention toward entrepreneurial behavior because they affect individuals’ needs and emotions (López-Núñez et al., 2022). Personality traits can interact with environmental variables and affect individual psychological behaviors (Lihua, 2021). Many studies on personality traits concentrated mainly on three infer traits: nAch, LoC, and ESE (Karabulut, 2016; Nasip et al., 2017; Uysal et al., 2022). Therefore, this study focuses on those three major factors as personality factors.

nAch as one of the personality traits concept related to entrepreneurial activity was introduced in the 1960s and 1970s (McClelland, 1961; Bandura, 1977). The nAch impacts one’s IntEnt (McClelland, 1961), defined as having one’s desire and ambition to succeed (Karabulut, 2016). Individuals with a high nAch tend to be diligent, tenacious, and determined. They feel more ability, are more self-confident, perform better, actively research in their environment, have less acceptance of failure, and have a higher power to triumph under difficult situations than those with a low nAch (McClelland, 1961, 1965, 1987). According to Kristiansen and Indarti (2004), the low nAch is linked with poor expectations, low competence and inspiration, a tendency toward self-blame, and an orientation toward failure. Therefore, it can be expected that the individuals with a high nAch strongly believe in their capacity to establish a new venture, controlling the events of establishing a process, higher ESE and IntEnt than others. The interaction of the nAch with other variables sometimes shows essential figures. Although other variables like LoC were affected by variables such as gender, nAch did not interact with such variables (Uysal et al., 2022). This raises the importance of testing the effect of nAch in the presence of other variables.

LoC is another personality trait first introduced by social learning theory in the 1950s (Rotter, 1954, 1966). It measures an individual’s belief in their ability to control the environment through their actions. An individual has a high LoC if that individual believes that they can affect the outcome of their actions out of their skills, abilities, and efforts (Karabulut, 2016; Uysal et al., 2022). Begley and Boyd (1987) noted that LoC and nAch have a genuine relationship as individuals with high nAch are persuaded by their high LoC that their actions will result in desired outcomes (Uysal et al., 2022). In this regard, Rauch and Frese (2007) state that specific personality factors related to entrepreneurial behavior, such nAch and LoC, are more helpful in predicting business success than other personality factors. Many studies revealed that high LoC is closely related to high levels of IntEnt (Sesen, 2013; Arkorful and Hilton, 2021); in contrast, some research produced different findings (Kristiansen and Indarti, 2004; Nasip et al., 2017). However, as mentioned by Mueller and Thomas (2001), LoC is closely related to cultural variations, where LoC orientation is higher in individualistic cultures than in collectivistic cultures. This may therefore result in more creative and innovative behaviors related to entrepreneurship. According to Ajzen (1991), the LoC and ESE are different, as the LoC refers to an individual’s beliefs that they have control over the results of their action. In contrast, ESE refers to the perceived ease or difficulty of performing specific behavior, in our case, doing business. The conflicting findings regarding LoC reveal the necessity of testing the effect on LoC in the presence of some mediating variables and other independent variables, as shown down.

ESE, as shown above, is a personality trait that refers to an individual’s belief in their capability to overcome complex activities such as the process of new venture creation (Boyd and Vozikis, 1994; Lihua, 2021). The term self-efficacy was derived from Bandura’s social-cognitive career theory to describe individuals’ belief in their capability to perform a particular action, “ability expectancy,” and the expectation that this action will produce the desired result, “outcome expectancy” (Bandura, 1986). Bandura (1977) adds that experience, learning, training, and social persuasion are significant antecedents that develop an individual’s ESE. Individuals choose self-employment as a career option because they have high ESE (Zhao et al., 2005). According to Hechavarria et al. (2012), individuals with high ESE work harder to meet their obligations, and they attribute failure to internal reasons under their control rather than the external environment (Bandura, 1982). Li et al. (2020) state that individuals with higher ESE prefer challenging tasks and show greater creativity, resilience, and optimism when establishing a successful business intention. Uysal et al. (2022) believe that high ESE is a prerequisite for entrepreneurial motivation. However, prior entrepreneurship studies investigating antecedents of ESE, such as personality traits and environmental and situational factors, stay rare (Boyd and Vozikis, 1994; Liguori et al., 2018; Memon et al., 2019). Thus, this study attempts to fill this research gap by investigating the mediating role of ESE in motivating students’ IntEnt in a developing country like Yemen.

### 2.3. Environmental factors

Many social-cognitive theories and empirical studies integrated personality traits and environmental factors to determine one’s IntEnt. For instance, Davidsson (1995) economic-psychological model, Lüthje and Franke (2003) model of IntEnt; Kristiansen and Indarti (2004) compared the influence of different economic and cultural contexts on students’ IntEnt; Wang et al. (2010) studied the effect a set of environmental and individual factors on one’s motivation to become an entrepreneur; Gerba (2012) and Sesen (2013) tested a comprehensive model of IntEnt that combines environmental factors and personality traits; Al-Qadasi et al. (2021) tested an integrated IntEnt model that combined TPB personality factors and LFM contextual factors. Following previous studies, environmental conditions can include several factors influencing one’s IntEnt. In this study, we are mainly interested in the most critical environmental factors, namely entrepreneurial finance, entrepreneurial social networks, and availability of business information as below.

Entrepreneurial finance is critical in establishing a new venture, particularly in the least developed countries (Kristiansen and Indarti, 2004). It is the most crucial factor in measuring the level of support for developing entrepreneurship in any economy. According to Sesen (2013) and Ma et al. (2019), many entrepreneurial ventures fail due to a lack of start-up business funding. There are different ways to fund budding entrepreneurs. For instance, self-funding the new venture (Cetindamar et al., 2012), getting a bank loan or credit (Ma et al., 2019), and establishing a university business incubator (Wonglimpiyarat, 2016). Also, one of the most popular ways of entrepreneurial funding is through entrepreneurial social networks (Liguori et al., 2018). In the study context, research on the entrepreneurship ecosystem indicates that the entrepreneurs’ first reliance on venture capital was their savings, followed by friends (Alshebami and Alzain, 2022). In the following subsection, we focus on the role of entrepreneurial social networks as crucial environmental factors regarding guiding and supporting budding entrepreneurs to be entrepreneurial as follows.

Entrepreneurial social networks are an essential resource for developing entrepreneurial activities (Ruiz-Palomino and Martínez-Cañas, 2021), where the perception of support affects career choice in general and particularly paths to business ownership (Edelman et al., 2016). Healthy entrepreneurial social networks can provide contacts and relationships to improve entrepreneurial capability building (Zhao et al., 2021), decrease business uncertainty (Kristiansen and Ryen, 2002), survive over time (De Carolis et al., 2009), access to venture capital (Liguori et al., 2018), and access to business information (Sesen, 2013).

Access to business information is another environmental factor associated with the ready availability of business information (Kristiansen and Indarti, 2004; Sesen, 2013). It is crucial for entrepreneurs who require market-related information before launching a new venture to define competitive conditions. Business information is used to make entrepreneurial decisions through a cognitive perspective that provides a better understanding of market opportunities through accurate and relevant information.

Generally, environmental factors are known under various names. Previous entrepreneurial literature grouped the three environmental factors mentioned above into Instrumental Readiness (e.g., Kristiansen and Indarti, 2004; Gerba, 2012; Memon et al., 2019). Following previous studies, the main environmental factors tested in this study were Instrumental Readiness (InsRead).

### 2.4. Situational factors

Besides the above discussion, this study reveals different situational factors influencing one’s decision to be an entrepreneur. Shapero (1982), Davidsson (1995), Kennedy et al. (2003), Arrighetti et al. (2016), and Yukongdi and Lopa (2017) contend that the process of making entrepreneurial decisions would not be independent of the situation in which the new entity will launch and operate. Shapero (1982) describes situational factors as changes in a life path that may lead to entrepreneurship; Lüthje and Franke (2003) state that situational factors influence the relationship between attitude and behavior, Arrighetti et al. (2016) conclude that situational factors as an obstacle to entrepreneurial activities may not affect one’s IntEnt but negatively influences the likelihood of launching a new venture. He classified them as external factors that pull or push individuals toward entrepreneurship. To better understand the influence of situational factors on one’s IntEnt, this study investigates the direct and indirect relationships between a set of situational factors and students’ intention to start a new business in a unique social-economic context, Yemen. Thus, we can assume that the current situation in the study context (political-economic crisis) and changes in it will be one of the most influential situational factors on one’s IntEnt decision. In this study, we mainly focus on three major situational factors, i.e., psychological, financial, and social, as well as one factor regarding the availability of resources during such uncommon circumstances. These four situational factors received lesser research attention than other situational factors in previous IntEnt literature (Mouselli and Khalifa, 2017). Thus, this study tries to fill this void.

#### 2.4.1. Current political-economic situation

Yemen is one of the developing countries located in the Middle East. The primary source of income for the government is natural Gas and Oil production. Since 2011, the country has sunk into a civil and regional war, causing several economic and political issues. Oil, Gas production, and almost all government and private economic activities have stopped, resulting failed of authorities to provide salaries for public-sector employees, which is only intermittently and partly paid since 2016. For the private sector, most operations and business activities have ceased due to the high cost of inputs and lack of supplies, as well as insecurity, leading to vast layoffs of the workforce, unemployment, and higher poverty (Alshebami and Alzain, 2022). According to the world bank group report, Yemen’s GDP contracted by 39% between 2014 and 2019 (World Bank, 2019). Before the current political-economic situation, Yemen was ranked number one out of 55 economics regarding the percentage of adults who think entrepreneurship is a good career choice (Stevenson et al., 2010). The study added that 60% of Yemeni adults believe they are qualified to start a business, 27% are ready to venture shortly, and 43% are afraid to fail. Although these findings are very enthusiastic to entrepreneurship as the better career choice, it is unclear whether this still holds in light of the current situation (Al-Jubari, 2019). Thus, our study focused on the influence of situational factors on students’ IntEnt and developed a theoretical framework.

From the previous discussion, we can see the importance of investigating the individual and mutual effects of the selected variables. Studying each variable separately can show different behavior to learning these variables as one set of factors. This study tried to fill this gap by the mutual and interactive effect of personality traits and environmental and situational factors on IntEnt.

### 2.5. Hypothesized model

**Figure 1:**
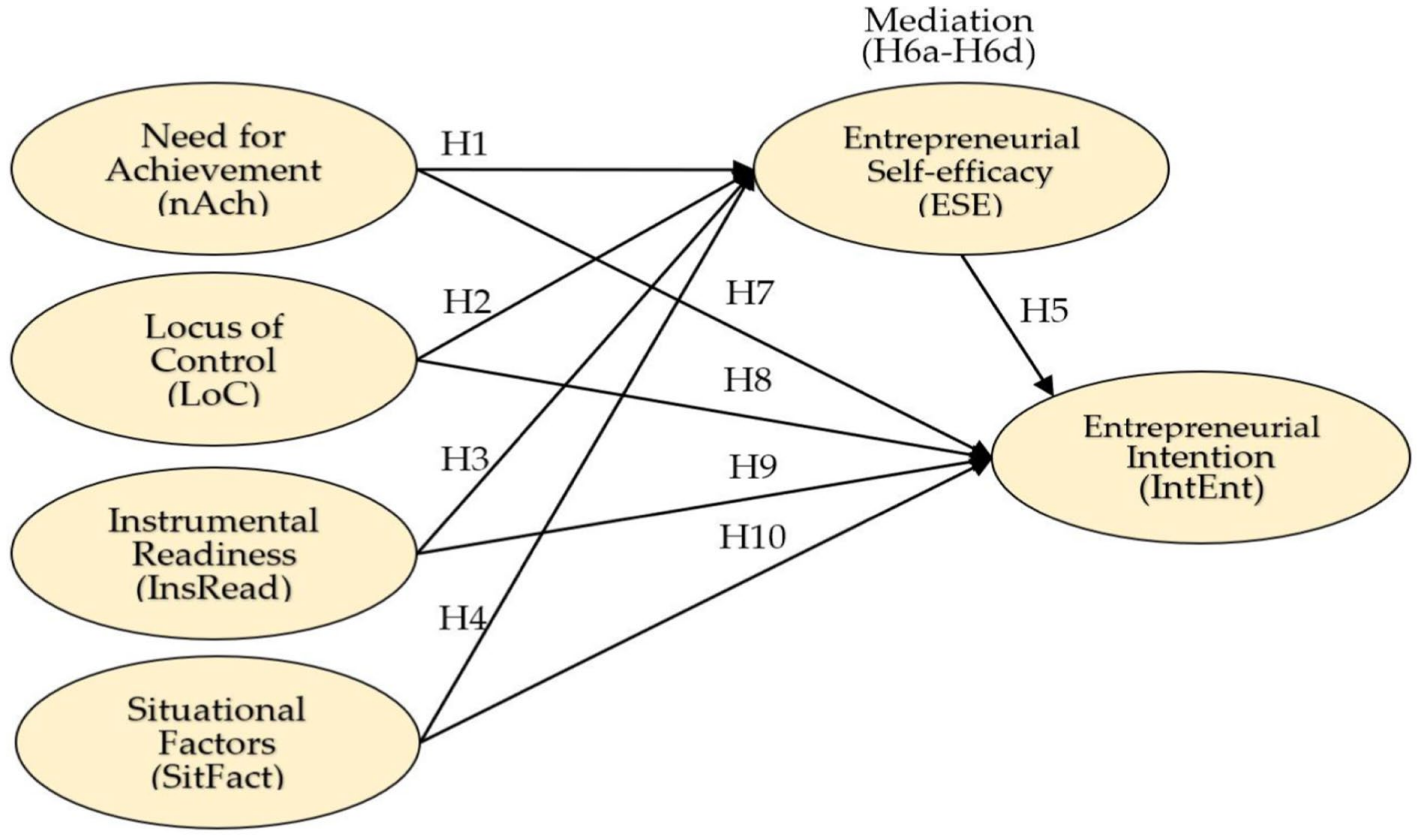
Conceptual framework and hypothesized relationships.

Increasing our knowledge and understanding of various interacting factors that influence an individual’s IntEnt might require combining the impact of a broad range of influential factors giving due consideration to crucial factors in the literature. As a unique contribution to the entrepreneurial behavior literature, this study elucidates how personality traits and environmental and situational factors interact to shape one’s IntEnt. This may help bridge the IntEnt gap and develop a comprehensive model of IntEnt with high predictive power. This study postulates a theoretical model that incorporates two significant predictors of personality traits (i.e., nAch and LoC), one predictor of environmental factors named InsRead (i.e., entrepreneurial finance, entrepreneurial social networks, and entrepreneur access to business information), and one predictor of situational factors (i.e., political-economic situation) to determine the factors influencing IntEnt among undergraduate students in two universities (public and private) in the unique socio-economic setting. The proposed model tests the direct and indirect influence of personality traits, InsRead, and situational factors (SitFact) on students’ IntEnt. ESE acts as a mediator variable to measure students’ entrepreneurial skills for the development of IntEnt. Figure 1 illustrates the conceptual model of the study and hypothesized relationships.

Finally, based on the theoretical foundation in the literature review above, a set of hypotheses have been developed to be empirically tested in this study, as shown in Table 1.

**Table 1:** Study hypotheses.

Hypo.	Description
*H1*	nAch has a direct effect on ESE
*H2*	LoC has a direct effect on ESE
*H3*	InsRead has a direct effect on ESE
*H4*	SitFact has a direct effect on ESE
*H5*	ESE has a direct effect on IntEnt
*H6a–d*	ESE mediates the nAch (*H6a*), LoC (*H6b*), InsRead (*H6c*), SitFact (*H6d*), and IntEnt relationship
*H7*	nAch has a direct effect on IntEnt
*H8*	LoC has a direct effect on IntEnt
*H9*	InsRead has a direct effect on IntEnt
*H10*	SitFact has a direct effect on IntEnt

## 3. Methodology

### 3.1. Participants and sample size

The participants of this study were college students in Yemen. To ensure the representativity and variability of respondents, the participants were selected from two universities (public and private), the oldest and most prominent institutes of higher education in the state. Concretely, 79,460 students in the public university and 10,729 students in the private university were actively enrolling in the 2019/2020 academic year. The study targeted final-year undergraduate students. According to Liñán and Chen (2009), in the domain of IntEnt research, student samples, particularly final-year students, are considered a suitable sampling strategy. The targeted population size of both universities was more than 16,000 undergraduate students last year. A stratified sampling technique was employed. According to Thompson (2012), if the study population is 20,000 or less, the recommended sample size is 376. A study (Kline, 2015) reports that there should be at least ten responses per parameter. The study questionnaire comprised 23 items. Thus, the sample size of this study meets prior conditions. A pilot study was conducted among ten students. The results were satisfactory. Therefore, the reliability and validity of the scale have been recognized. According to Julious (2005), the recommended sample size of a pilot study is 12 and could be reduced to 10. Thus, the sample size of the pilot study was suitable. A total of 750 questionnaires were distributed at different locations within the two university campuses. The data for self-administered questionnaires were provided by 487 final-year students, with a response rate of 64.93%. Of these, (71.77%) were male, and (28.33%) were female, with an average age of 24.11 years old (SD = 1.20). The structure of the sample by age, gender, and the university is shown in Table 2.

**Table 2:** The structure of the sample.

Dimensions	Category	Frequency (N)	Frequency (%)
Age	Less than 24	181	37.20
	24–25	232	47.60
	≥26	74	15.20
Gender	Male	349	71.77
	Female	138	28.33
University	Public	363	74.50
	Private	124	25.50

### 3.2. Measures

IntEnt was a dependent variable measured using a four-item scale based on the prior work of Liñán and Chen (2009). A sample item is “I am ready to do anything to become an entrepreneur.”

Personality traits: nAch and LoC were measured through four-item for each scale as utilized by Kristiansen and Indarti (2004) and Mueller and Thomas (2001). Sample items are “I will do very well in fairly difficult tasks relating to my study and my work” and “Diligence and hard work usually lead to success.” ESE was a mediate variable measured with a four-item scale adapted from Liñán and Chen (2009). A sample item is “Opening and operating a business is easy for me.”

InsRead was measured mainly based on environmental supporting factors. Based on the works of Kristiansen and Indarti (2004), Gerba (2012), and Memon et al. (2019), three-item were used to measure this scale. These items are “I have access to capital to start to be an entrepreneur,” “I have good social networks that can be utilized when I decide to be an entrepreneur,” and “I have access to supporting information to start to be an entrepreneur.”

SitFact was measured using four items developed by Mouselli and Khalifa (2017) to measure the impact of the political-economic crisis on students’ IntEnt in the Syrian context. We agree with their idea that there are three items regarding the crisis that will affect an individual’s psychological, financial, and social status. A sample item is “crisis affected my psychological situation.” The fourth item was developed to measure the availability of resources during the political-economic crisis “The current political-economic crisis has restricted resources that are necessary to start up business.”

All items were measured using a 5-point Likert scale ranging from “1 = strongly disagree” to “5 = strongly agree.” The measures were developed in English and then translated into Arabic, the local language of the respondents. The translation was done and revised by three different professionals.

### 3.3. Data screening

To guarantee that the data collected were clean and suitable for analysis. *First*, the missing values should be examined. It was observed that a few accounts of the responses had missing values, which was less than the threshold of 10% of the responses; to be exact, 7% were missing data. Expectation Maximization (EM) method was used to replace the missing data through (SPSS) version 22. *Second*, for the normality distributed issue, according to (Hair et al., 2022), one advantage of using PLS-SEM is that there is no necessity for a normality test. Thus, normality distributed issue was not a concern. The *third* validity and reliability examination that should be reported is common method bias (CMB). We checked CMB to ensure that the data did not suffer from biased. Using exploratory factor analysis (EFA), all the key variables were entered to see whether a single factor could account for significant covariance. CMB was tested by Harman’s one-factor test (Podsakoff et al., 2003). The result showed that a single-factor structure explains (29.229%) of total variances, lower than 50% (Podsakoff et al., 2003). Thus, the CMB was not a concern in this study Table 3. All the Variance inflation factor (VIF) values were obtained in the inner model from complete collinearity statistics below the threshold level of 3.3. (Kock, 2015).

**Table 3:** Total variance explained (Harman’s single-factor test).

Factor	Extraction sums of squared loadings		
Component	Total	% of variance	Cumulative %
1	7.430	29.229	29.229

Extraction method: Principal axis factoring.

To check the predictive and effect size, the researcher used cross-validated redundancy (*Q*^2^) and R-squared correlation coefficients (*R*^2^). The value of *R*^2^ is the variance that describes all independent constructs. According to Cohen (1988), an *R*^2^ value between 0.02 and 0.13 is weak, 0.13–0.26 is moderate, and more than 0.26 is strong. In this study, the *R*^2^ values of ESE and IneEnt are strong. Furthermore, *Q*^2^ is evaluated to estimate the overall accuracy of the study model. According to Henseler et al. (2015), for *Q*^2^ to be valid, its value must be greater than zero. In this study, the *Q*^2^ values of ESE and IneEnt are more significant than zero. Table 4 shows that the value of *R*^2^ and *Q*^2^ meet the validity evaluation criteria, suggesting that the data are ready for further analysis.

**Table 4:** *R*^2^ and *Q*^2^.

Constructs	*R* ^2^	*R*^2^ adjusted	*Q* ^2^
ESE	0.450	0.445	0.435
IntEnt	0.370	0.364	0.329

ESE, entrepreneurial self-efficacy; IntEnt, entrepreneurial intentions.

### 3.4. Data analysis

The collected data were statistically analyzed utilizing the SmartPLS version 4.0, which employs the Partial Least Squares Structural Equation Modeling (PLS-SEM) approach. For a number of reasons, PLS-SEM is a better approach for this study than Covariance-based Structural Equation Modeling (CB-SEM). First, the nature of the study is an exploratory study, thus PLS-SEM is more appropriate than the BC-SEM approach. Second, the study is designed for examining mediation relationships between the variables included in the study model; PLS-SEM is fit for examining such relationships. Third, as mentioned above, distribution assumptions are not relevant to PLS-SEM. Hair et al. (2022), reported that the PLS-SEM employs a two-step approach. First is assessments of the measurement model or outer model. The second is the structural model or inner model. The measurement model measures the relationships between the observed and their latent variables, whereas the structural model measures the relationships between the latent variables. The results section below discusses the outcome of applying these two techniques methods and hypotheses testing results.

## 4. Results

### 4.1. Assessments of the measurement model

The model assessment focuses on the measurement models to assess the construct’s convergent validity. It includes three indices. First is factor loading, individual indicator reliability, which should be above the critical threshold of 0.70. The second is composite reliability (CR), the score should be above 0.70. The third is average variance extracted (AVE), the value should be above 0.50 (Hair et al., 2022). Table 5 shows the findings of assessments of the measurement model, indicating that the indicators’ outer loadings (standardized factor loading) ranged between 0.723 and 0.907. One item was deleted from the LoC scale due to low loadings score (i.e., LoC3 = 0.658). The AVE ranges between 0.637 and 0.765 on each scale. The CR values are above the threshold of 0.70 and range between 0.755 and 0.0.860 on each scale. Lastly, Cronbach’s alpha coefficient reliability values for all scales were acceptable and above 0.749 in each case, indicating the scale’s convergent validity.

**Table 5:** Convergent validity and reliability.

Factors/scales	Items	SFL	AVE	CR	Cronbach’s α
Entreprenurial intentions	IntEnt1	0.842	0.692	0.858	0.852
	IntEnt2	0.866			
	IntEnt3	0.806			
	IntEnt4	0.811			
Need for achievement	nAch1	0.836	0.690	0.857	0.851
	nAch2	0.819			
	nAch3	0.823			
	nAch4	0.844			
Locus of control	LoC1	0.842	0.665	0.755	0749
	LoC2	0.778			
	LoC4	0.825			
Entrepreneurial self-efficacy	ESE1	0.836	0.687	0.859	0.847
	ESE2	0.739			
	ESE3	0.876			
	ESE4	0.858			
Insturmental readiness	InsRead1	0.907	0.765	0.860	0.848
	InsRead2	0.815			
	InsRead3	0.899			
Situational factor	SitFact1	0.723	0.637	0.857	0.818
	SitFact2	0.815			
	SitFact3	0.837			
	SitFact4	0.814			

SFL, standardized factor loading; AVE, average variance extracted; CR, composite reliability; Item (LoC3) was deleted due to low loading.

Two measures, variable correlation (Root square of AVE) and cross-loadings, should be reported for discriminant validity assessment. Table 6 shows the results of the discriminant validity coefficients (Fornell-Larcker criterion) and Heterotrait-monotrait (HTMT) ratios for measurement model assessment. The findings indicate that all the studied scales’ reliability and convergent validity criteria were met, as suggested by Hair et al. (2022). To be exact, the square root of AVE for each scale was more significant than the correlations with other scales included in the study model, and the HTMT ratio values meet the threshold of <0.85.

**Table 6:** Discriminant validity coefficients (fornell-larcker criterion) and heterotrait-monotrait (HTMT) ratios.

Scales	nAch	LoC	InsRead	SitFact	ESE	IntEnt
nAch	***0.831***	0.833	0.126	0.155	0.692	0.622
LoC	0.671	***0.816***	0.188	0.099	0.765	0.627
InsRead	0.110	0.152	***0.875***	0.097	0.234	0.059
SitFact	0.054	0.010	−0.066	***0.798***	0.147	0.171
ESE	0.594	0.606	0.212	−0.054	***0.829***	0.552
IntEnt	0.536	0.506	0.047	0.156	0.478	***0.832***

Italic and bold values represent the square roots of AVEs. The below values represent the squared correlations. The above values represent HTMT ratios. nAch, need for achievement; LoC, locus of control; InsRead, instrumental readiness; SitFact, situational factors; ESE, entrepreneurial self-efficacy; IntEnt, entrepreneurial intentions.

### 4.2. Assessment of structural model

**Figure 2:**
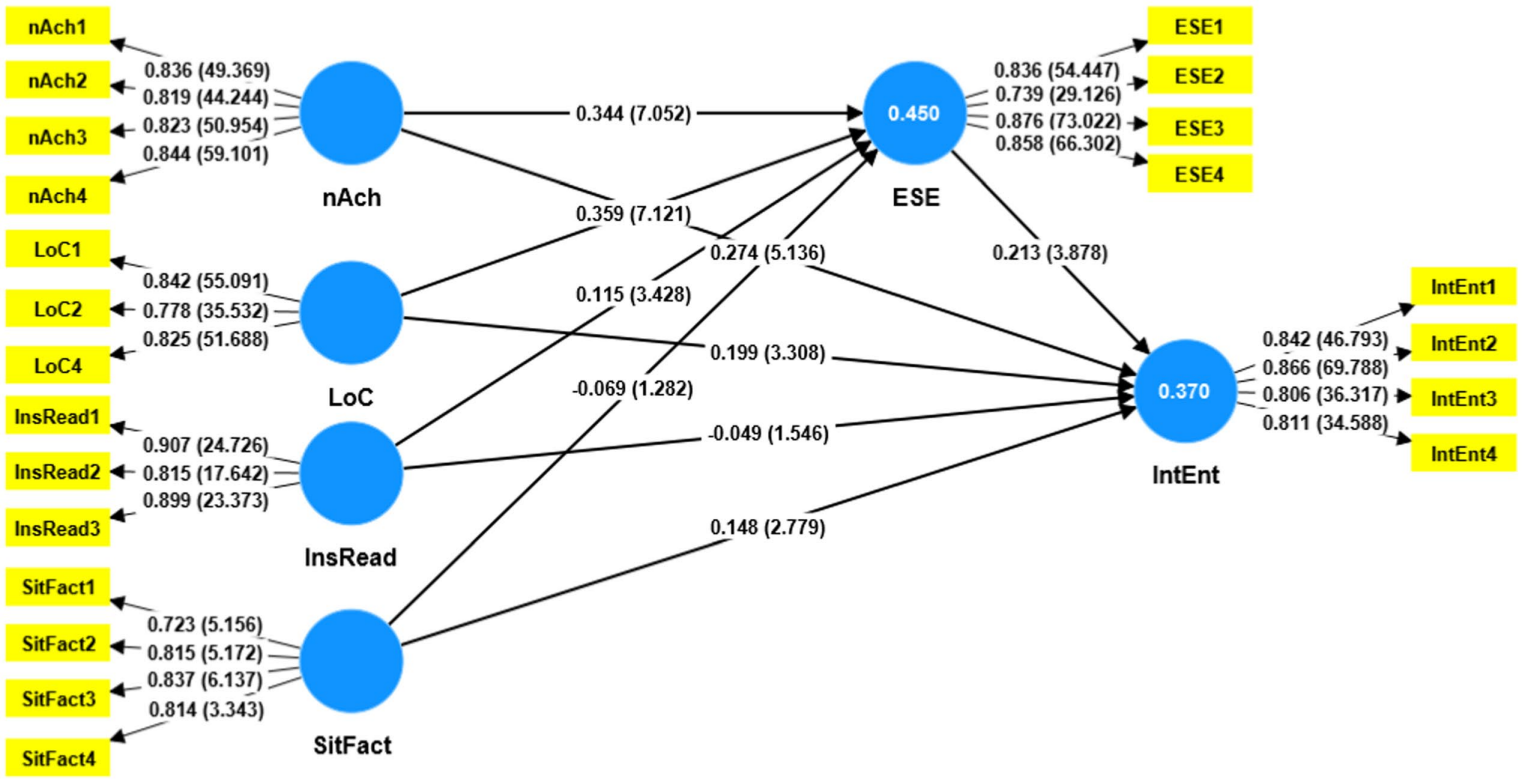
Structural model.

After confirming the reliability and validity of construct measures, the next step is assessing the structural model. This step includes testing the model’s ability in explaining the exogenous constructs and the relationships between the study variables. More specifically, the model’s ability is investigated by assessing the squared correlations between the exogenous variables (i.e., nAch, LoC, InsRead, SitFact, and ESE), predicting the endogenous latent variable under consideration (i.e., IntEnt) or *R*^2^ and the *t*-values. The path coefficient of the study model and the standardized outer loadings for the reflective measurement models are shown in Figure 2. The result of the *R*^2^ value was moderate = 0.370 (Chin, 1998), which means that the exogenous variables can explain approximately 37% of the variances in the IntEnt.

Part of the structural model assessment is testing Q-square and F-square. Q-square measure the predictive relevance of the model, while F-square measures the change in *R*^2^ when the exogenous variables are removed. *Q*^2^ tests showed scores that were much higher than the threshold of zero. This indicates that the model has established predictive relevance of the dependent variables. *F*^2^ scores also show satisfactory levels. Most of the *F*^2^ scores are higher than the threshold of 0.02. Only two scores are less than 0.02. These results are shown in Tables 7, 8.

**Table 7:** *Q*^2^ predict test.

	*Q*^2^ predict	RMSE	MAE
IntEnt	0.329	0.826	0.599
ESE	0.434	0.756	0.597

IntEnt, entrepreneurial intentions; ESE, entrepreneurial self-efficacy.

**Table 8:** *F*^2^ predict test.

	IntEnt	ESE
IntEnt		
ESE	0.039	
InsRead	0.004	0.023
LoC	0.031	0.127
nAch	0.059	0.118
SitFact	0.034	0.009

IntEnt, entrepreneurial intentions; ESE, entrepreneurial self-efficacy; nAch, need for achievement; LoC, locus of control; InsRead, instrumental readiness; SitFact, situational factors.

### 4.3. Hypothesis testing

As a general rule, for the significance testing, the resulting empirical *t-*value should be greater than 1.96 at a significance level of 0.05. Our study applies the recommendation from Hair et al. (2022), where a total of 5,000 subsamples were extracted for bootstrapping settings to confirm the significance of all path relationships in the structural model, *t*-values, and hypotheses testing at a significance level of 0.05. The *t*-values for each scale exceeded 1.96, confirming the significance of the relationships between the study scales, except the *t*-values for InsRead → IntEnt, SitFact → ESE, and SitFact → ESE → IntEnt were 1.54, 1.28, and 1.194, respectively. The structural model results suggested proceeding with further analysis to determine the direct and indirect relationship between the study variables and hypothesis testing as follows:

#### 4.3.1. Direct hypotheses testing

Table 9 and Figure 2 show the direct relationships between the study scales and hypotheses testing results. For the hypothesized path relationships between personality traits (nAch and LoC), InsRead, SitFact, and ESE, it was found that full support for nAch, LoC, and InsRead but not SitFact. More especially, path coefficients of personality traits, i.e., nAch → ESE (*β* = 0.334, *t* = 7.052, *p* = 0.000), LoC → ESE (*β* = 0.359, *t* = 7.121, *p* = 0.000), and InsRead, i.e., InsRead → ESE (*β* = 0.115, *t* = 3.428, *p* = 0.000) were significant but not path coefficient of SitFact, i.e., SitFact → ESE (*β* = −0.069, *t* = 1.282, *p* = 0.200). Thus, *H1*, *H2*, and *H3* were supported but not *H4*. A full support relationship was found between ESE and IntEnt, i.e., ESE → IntEnt (*β* = 0.213, *t* = 3.878, *p* = 0.000). Thus, *H5* was supported. For the direct hypothesized paths coefficient from personality traits (nAch and LoC), InsRead, SitFact to IntEnt, it was found that full support for nAch, Loc, and SitFact but not InsRead. To be exact, personality traits, i.e., nAch → IntEnt (β = 0.274, *t* = 5.136, *p* = 0.000), LoC → IntEnt (*β* = 0.199, *t* = 3.308, *p* = 0.001), and SitFact, i.e., SitFact → IntEnt (*β* = 0.148, *t* = 2.779, *p* = 0.005) were significant but not path coefficient of IstRead, i.e., InsRead → IntEnt (*β* = −0.049, *t* = 1.546, *p* = 0.122). Thus, *H7*, *H8*, and *H10* were supported but not *H9*. Based on the results shown in Tables 5, a strong positive significant relationship was found between personality traits (LoC and nAch) to ESE (*β* = 0.359) and (*β* = 0.334), respectively, followed by nAch and ESE to IntEnt (*β* = 0.274) and (*β* = 0.213), respectively, indicating the strong direct effect such personality traits in developing students’ IntEnt.

**Table 9:** Direct hypotheses testing.

Hypo.	Path	Estimates *β* value	SE	*T* value	value of *p*	Findings
*H1*	nAch → ESE	0.334	0.049	7.052	0.000	Supported
*H2*	LoC → ESE	0.359	0.050	7.121	0.000	Supported
*H3*	InsRead → ESE	0.115	0.033	3.428	0.000	Supported
*H4*	SitFacrt → ESE	−0.069	0.054	1.282	0.200	Not Supported
*H5*	ESE → IntEnt	0.213	0.055	3.878	0.000	Supported
*H7*	nAch → IntEnt	0.274	0.053	5.136	0.000	Supported
*H8*	LoC → IntEnt	0.199	0.060	3.308	0.001	Supported
*H9*	InsRead → IntEnt	−0.049	0.032	1.546	0.122	Not Supported
*H10*	SitFact → IntEnt	0.148	0.053	2.779	0.005	Supported

nAch, the need for achievement; LoC, the locus of control; InsRead, instrumental readiness; SitFact, situational factors; ESE, entrepreneurial self-efficacy; IntEnt, entrepreneurial intention.

#### 4.3.2. Mediation hypotheses testing

In this investigation, ESE was hypothesized to mediate between personality traits (nAch and LoC), InsRead, SitFact, and IntEnt (i.e., *H6*a–*H6*d). As shown in Table 10, the path coefficients of nAch → ESE → IntEnt (*β* = 0.073, *t* = 3.593, *p* = 0.000), LoC → ESE → IntEnt (*β* = 0.076, *t* = 3.176, *p* = 0.002), and InstRead → ESE → IntEnt (*β* = 0.024, *t* = 2.560, *p* = 0.011) supported mediation hypotheses, *H6*a, *H6*b, and *H6*c but not *H6*d, i.e., SitFact → ESE → IntEnt (*β* = −0.015, *t* = 1.194, *p* = 0.233), which indicates that ESE partially mediated the relationship between nAch, LoC, InsRead, and IntEnt. SitFact was not mediated by ESE but had a positive direct effect on IntEnt. For this reason, the findings regarding the impact of mediation variables lead us to accept *H6*a–*H6*c but not *H6*d.

**Table 10:** Mediation effects of nAch, LoC, InsRead, and sitfact on ESE to IntEnt relationship.

Hypo.	Path	Estimates *β* value	SE	*T* value	value of *p*	Findings
*H6*a	nAch → ESE → IntEnt	0.073	0.020	3.593	0.000	Supported
*H6*b	LoC → ESE → IntEnt	0.076	0.024	3.176	0.002	Supported
*H6*c	InsRead → ESE → IntEnt	0.024	0.010	2.560	0.011	Supported
*H6*d	SitFact → ESE → IntEnt	−0.015	0.012	1.194	0.233	Not supported

nAch, need for achievement; LoC, locus of control; InsRead, instrumental readiness; SitFact, situational factors; ESE, entrepreneurial self-efficacy; IntEnt, entrepreneurial intention.

## 5. Discussion

The study’s primary objective was to assess the influence of certain personality traits and environmental and condition factors on students’ IntEnt. ESE is mediating in the relationship between these three groups of factors and IntEnt. The significance of this study comes from Shapero (1982), Davidsson (1995), Lüthje and Franke (2003), and others, who highlighted the importance of interaction between personality, environmental, and situational factors in measuring an individual’s IntEnts. Similarly, Arrighetti et al. (2016) provide evidence that measuring IntEnt is effective when factors such as personality and environmental as well as situational are investigated, and notions such as “necessity-based” and “opportunity-based” might be a driver of entrepreneurship in challenging contexts (Devece et al., 2016). This is particularly so in the current study context. The study model assumed that variables such as personality traits, environment, and situational factors play a crucial role in determining individuals’ IntEnt (Yukongdi and Lopa, 2017; Anjum et al., 2020; Al-Qadasi et al., 2021). The model’s explanatory power improved by including ESE as mediating variable to 37%. This opens the way for more investigations using such interaction between personality traits and contextual factors in IntEnt research.

As one part of the objective of this study, we posited that factors such as personality traits, i.e., nAch and LoC influence students’ IntEnt and are mediated by ESE. The findings of prior empirical studies were inconsistent related to the relationship between personality traits such as nAch and LoC and IntEnt. Although Çolakoğlu and Gözükara (2016) and Vodă and Florea (2019) found nAch and LoC motivate students’ IntEnt, Kristiansen and Indarti (2004) did not find similar results; they found both personality traits nAch and LoC do not influence students’ IntEnt in Norway and Indonesia context. Along the same line, Nasip et al. (2017) and Yukongdi and Lopa (2017) found that nAch has a significant influence on Malaysian and Thailand students’ IntEnt but not LoC. In contrast, Sesen (2013) found that LoC significantly influences students’ IntEnt in Turkey but not nAch, suggesting that the personality traits differ across countries, cultures, and between men and women (Uysal et al., 2022). This research investigates personality traits: nAch and LoC as antecedent factors that influence students’ IntEne directly and indirectly *via* mediating role of ESE. The direct relationship between nAch and LoC and IntEnt were statistically significant (*β* = 0.274***) and (*β* = 0.199***), respectively (Hypotheses 7 and 8). The indirect relationship between nAch and LoC and IntEnt *via* ESE was significant (*β* = 0.073***) and (*β* = 0.076***), respectively (Hypotheses 6a and b). According to Begley and Boyd (1987), a high nAch has consonant with LoC (Uysal et al., 2022). The LoC persuades individuals with a high nAch to do actions that lead to desired outcomes (Rauch and Frese, 2007). He adds that the nAch and LoC are more helpful in predicting an individual’s IntEnt than the other personality traits. The direct relationships between nAch and LoC and ESE were also significant (*β* = 0.334***) and (*β* = 0.359***), respectively (Hypotheses 1 and 2). These findings align with the previous work of Uysal et al. (2022), who indicated a positive and significant relationship between personality traits (nAch and LoC) and ESE, and the ESE mediated the relationship between LoC and nAch and IntEnt.

One key determinant of the emergence of budding entrepreneurs in any economic, advanced, or unadvanced, is a supportive environment. This study mainly focused on three major environmental factors: entrepreneurial finance, entrepreneurial social networks, and access to business information. Like prior studies (Kristiansen and Indarti, 2004; Gerba, 2012; Memon et al., 2019), we called these three groups of factors InsRead. The study postulated a relationship between InsRead and students’ IntEnt. Suppose students realize a supportive environment for business creation, such as facility access to capital, availability of business information, and social networking resources. In that case, they will be more likely to have strong IntEnt (Al-Qadasi et al., 2021). However, the findings of the study did not support this idea. The direct influence of InsRead was insignificant and negative on students’ IntEnt (*β* = −0.049; Hypothesis 9). At the same time, the immediate effect of InsRead on ESE was a positive significant (*β* = 0.115***; Hypothesis 3). The indirect influence of InsRead on IntEnt *via* ESE was also positively significant (*β* = 0.024**; Hypothesis 6c). This means ESE mediates the relationship between InstRead and IneEnt. The entrepreneurial literature includes numerous studies demonstrating an insignificant correlation between InsRead and IntEnt (Gerba, 2012; Yukongdi and Lopa, 2017; Zhao et al., 2021). One reason for the negligible influence of InsRead may be the context of the current study, as bad political-economic conditions can negatively influence any business opportunity by affecting infrastructure and financial obstacles (Aldairany et al., 2018). To further understand this relationship, this study examined the influence of situation factors—political-economic situation—on students’ IntEnt.

While the majority of previous studies focused only on the effect of personality traits and environmental factors on an individual’s decision to engage in entrepreneurial action and become an entrepreneur (Byabashaija and Katono, 2011), this study design enabled an examination of whether situational factors—political-economic state—influence business creation. Economic and geopolitical instability creates unusual circumstances in societies, such as high youth unemployment, uncertain employment future, and the decline in the economy in general. According to Kennedy et al. (2003), unemployment and future commitments are considered major situational factors leading to business start-ups (Byabashaija and Katono, 2011). Thus, a specific situation can trigger the decision to start a business venture. The findings of this study revealed a significant direct relationship between SitFact and IneEnt (*β* = 0.148***; Hypothesis 10). Yemeni students treated the current political-economic situation as an opportunity instead of a threat. Moreover, they thought of entrepreneurial activities as their best future choice because of the high unemployment rate resulting from the current economic-political situation. This means that SitFact motivates individuals (university students) to engage in entrepreneurial activity and has not harmed their IntEnt in the study context. This finding contradicts a similar study done in the Syrian setting (Mouselli and Khalifa, 2017).

Nevertheless, the impact of the political-economic situation on IntEnt varies widely across contexts, and it depends on a set of interacting variables in each case, such as the type of political-economic crisis, a decline in economic opportunities, the hamper overall economic activities, and the cause emerging from the entrepreneurship literature. However, the direct relationships between SitFact and ESE, as well as the indirect effect of SitFact on IntEnt *via* ESE, were minor negative (*β* = −0.069) and (*β* = −0.015), respectively (Hypothesis 4) and (Hypothesis 6d). This issue is still a lacuna in entrepreneurship literature that should be investigated in future studies.

### 5.1. Theoretical implications

The findings of this research have implications for IntEnt theories and entrepreneurship development policy. The first theoretical contribution affirms that theories such as TPB, EE, Davidsson’s model, and LFM are helpful for understanding individuals’ IntEnt (Nabi et al., 2010). The study model tests the interaction of a set of personal, environmental, and situational factors that influence IntEnt; this will inspire future studies to add more variables as antecedents to the IntEnt models (Davidsson, 1995). Second, investigating the interaction between these three groups of factors and an individual’s IntEnt in a context with limited resources could uncover significant findings (Al-Qadasi et al., 2021). Third, the direct effect of personality traits and SitFact on students’ IntEnt were positive and significant but not InsRead. The indirect influence of personality traits and InsRead on students’ IntEnt through ESE were positive and effective, but SitFact was negative and insignificant. Thus, these findings provide novel empirical evidence for these relationships, which have important implications for the literature. Fourth, this study used ESE as a mediation variable to measure students’ IntEnt. Few studies have investigated ESE’s mediating role in the relationship between personality traits, environmental and SitFact, and IntEnt (Uysal et al., 2022). Thus, this study contributes to the entrepreneurial behavior literature by maintaining the importance of ESE for IntEnt. Finally, this study was carried out in Yemen, which has witnessed one of the world’s worst political-economic crises, high unemployment, and business uncertainty. Very few studies tested a comprehensive model on IntEnt in this region (Al-Jubari, 2019).

### 5.2. Practical implications

Besides the theoretical implications, this contribution provides valuable insights into policymakers, academicians, and other stakeholders. First, the results indicate that personality traits nAch and LoC are more relevant than InsRead in determining students’ IneEnt in the study context. Thus, academicians can develop university curriculums that create a tool to advance students’ personality traits. This would help to make a higher level of IntEnt even in case of the existence of unfavorable InsRead. Second, InsRead was shown to have a negative and insignificant direct influence on IntEnt while at the same time having a positive and significant indirect effect on IntEnt through ESE. University’s entrepreneurial ecosystem, such as entrepreneurial education and training programs, can enhance ESE, influencing students’ entrepreneurship and encouraging them to launch their business ventures. Third, SitFact was found to have a positive and significant direct influence on IntEnt, but it has a negative and insignificant indirect influence on IntEnt through ESE. Thus, government and non-government institutions should contribute to creating an entrepreneurial ecosystem that encourages IntEnt. Government and non-government institutions should provide higher opportunities for entrepreneurs to access capital, availability of business information, infrastructural, and political and economic support. This support can help encourage more entrepreneurs to establish and start new businesses.

### 5.3. Limitations and future research

This study has limitations that offer opportunities for future research. First, the research data were collected through a survey questionnaire method. According to Manstead (2018), the survey questionnaire method is an appropriate strategy for psychological research as it describes one’s state that affects their behavior. All questionnaire indicators were assessed. Most of the necessary precautions were taken, such as ruling out the possibility of CMV, and the study was based on a quantitative research approach using non-probability sampling. This is regarded as a study limitation. In line with previous contributions, this study has many limitations that should be considered in future research using such factors. The second limitation of this study is the sample size. Although the findings relate to final-year undergraduate students from two universities (public and private) in the same city, future research should be expanded to the other universities in the state to obtain a clearer image of the influence of personality, contextual, and situational factors on the IntEnt among post and undergraduate students in Yemen. Third, entrepreneurship may be influenced by different environmental and situational factors in countries with economic-political instability. Thus, future research should overcome the constraints of assessing these factors and determine more accurate measurements. Fourth, since this study is cross-sectional, future studies should be longitudinal to generate more accurate results regarding IntEnt in Yemen. Finally, future research should focus on the role of entrepreneurship education in shaping students’ IntEnt. Consequently, attitude toward entrepreneurship education could help better understand students’ attitudes toward entrepreneurship and IntEnt.

## 6. Conclusion

This study examines personality, environmental, and situational factors that may directly influence students’ IntEnt and indirectly through ESE in the Yemen setting. The study findings found that personality factors: nAch and LoC, play a significant role in affecting students’ IntEnt. Based on the study findings, the direct influence of InsRead on students’ IntEnt was negative and insignificant in the Yemen context. Still, the indirect effect *via* ESE was positive and significant. This study has revealed that the SitFact, economic-political instability condition, have motivated students’ IntEnt and did not harm their intentions to become future entrepreneurs. The direct influence of SitFact on IntEnt was significant. The indirect effect of SitFact on IntEnt *via* ESE was negatively insignificant. Considering the significance of personality traits, the influence of InsRead and SitFact on an individual’s IntEnt is subject to debate. The contribution of this study to entrepreneurial behavior literature by the application of Ajzen (1991) TPB, Shapero (1982) EE, Davidsson (1995) economic-psychological model, and Lüthje and Franke (2003) LFM in a unique context to choose entrepreneurship as a career path for college students after graduation. Thus, this study provides a better understanding of how personality traits and environmental and situational factors influence intentions of new business creation. The authors hope this study’s findings will inspire future studies to develop more rigorous measures to measure the environmental and situational factors that influence an individual IntEnt in societies suffering from political-economic instability.

## Data availability statement

The raw data supporting the conclusions of this article will be made available by the authors, without undue reservation.

## Author contributions

All authors contributed to the study. Conceptualization, NA and MAA; introduction, NA and ASA; literature review, NA and MAA; methodology, NA and MAA; Data collection and analysis, NA; investigation, NA; Final draft review and editing, AA; conceived the draft, supervision, and funding acquisition, GZ. All authors have read and agreed to the published version of the manuscript.

## Funding

The publication fee of this research was funded by Jilin University.

## Conflict of interest

The authors declare that the research was conducted in the absence of any commercial or financial relationships that could be construed as a potential conflict of interest.

The handling editor MM declared a past co-authorship with the author ASA.

## Publisher’s note

All claims expressed in this article are solely those of the authors and do not necessarily represent those of their affiliated organizations, or those of the publisher, the editors and the reviewers. Any product that may be evaluated in this article, or claim that may be made by its manufacturer, is not guaranteed or endorsed by the publisher.
